# Evidence for contamination as the origin for bacteria found in human placenta rather than a microbiota

**DOI:** 10.1371/journal.pone.0237232

**Published:** 2020-08-10

**Authors:** Rémi Gschwind, Thierry Fournier, Sean Kennedy, Vassilis Tsatsaris, Anne-Gaël Cordier, Frédéric Barbut, Marie-José Butel, Sandra Wydau-Dematteis

**Affiliations:** 1 Université de Paris, INSERM UMR-S 1139 (3PHM), Paris, France; 2 Hospital-University Department Risks in Pregnancy, Paris, France; 3 PremUp Foundation, Paris, France; 4 Department of Computational Biology, Institut Pasteur, USR 3756 CNRS, Paris, France; University of Wisconsin - Madison, School of Veterinary Medicine, UNITED STATES

## Abstract

Until recently the *in utero* environment of pregnant women was considered sterile. Recent high-sensitivity molecular techniques and high-throughput sequencing lead to some evidence for a low-biomass microbiome associated with the healthy placenta. Other studies failed to reveal evidence for a consistent presence of bacteria using either culture or molecular based techniques. Comparing conflicting “placental microbiome” studies is complicated by the use of varied and inconsistent protocols. Given this situation, we undertook an evaluation of the *in utero* environment sterility using several controlled methods, in the same study, to evaluate the presence or absence of bacteria and to explain contradictions present in the literature. Healthy pregnant women (n = 38) were recruited in three maternity wards. Placenta were collected after cesarean section with or without Alexis^®^ and vaginal delivery births. For this study we sampled fetal membranes, umbilical cord and chorionic villi. Bacterial presence was analyzed using bacterial culture and qPCR on 34 fetal membranes, umbilical cord and chorionic villi samples. Shotgun metagenomics was performed on seven chorionic villi samples. We showed that the isolation of meaningful quantities of viable bacteria or bacterial DNA was possible only outside the placenta (fetal membranes and umbilical cords) highlighting the importance of sampling methods in studying the *in utero* environment. Bacterial communities described by metagenomics analysis were similar in chorionic villi samples and in negative controls and were dependent on the database chosen for the analysis. We conclude that the placenta does not harbor a specific, consistent and functional microbiota.

## Introduction

The *in utero* sterility dogma posits that the fetus matures in a bacteria-free environment prior to birth. This dogma has been challenged by several publications suggesting the presence of bacteria in the fetal environment including inside the placenta [1–7]. Studies using combinations of quantitative PCR (qPCR), fluorescence *in situ* hybridization (FISH) and next-generation sequencing techniques propose the existence of a “placental microbiome” [1, 2, 8–18]. More generally, bacterial species were found in placenta, amniotic fluid, fetal gut, meconium and the neonate’s mouth, suggesting a microbial colonization of the fetus [1, 15, 19]. Interestingly, reports indicated that variations in the bacterial composition of the “placental microbiome” were associated with pregnancy outcomes such as preterm birth, chorioamnionitis, macrosomia, gestational diabetes mellitus, excess gestational weight gain, low birth weight, and pre-eclampsia [2, 8, 9, 11, 17, 18, 20–23]. However, the physiological role of such “placental microbiome” remains unknown.

Whereas a consensus exists on the low biomass of such a bacterial community compared to other body sites, the suggested composition of any placental microbiota remains variable across studies. Indeed, some researchers found that the bacterial communities in placental samples were indistinguishable from negative controls [24–28]. These contradictory results call into question the existence of a “placental microbiome” as well as the existence of a microbiota in amniotic fluid [29–31]. Perhaps one of the strongest arguments against a functional placental microbiota and its implied *in utero* colonization of the fetal intestine is our ability to readily generate germ-free mammals, primarily mice, in the laboratory. Supporting this idea is the fact that the fetal gut was recently proven to be sterile in an ovine model [32].

Today, researchers keep publishing studies defending or contesting existence of such “placental microbiome”, showing that the debate is not closed. Our hypothesis is that variability between studies arises from the use of several sampling and analysis techniques, and the variable presence of appropriate negative controls. Indeed, such low microbial biomass can be extremely sensitive to sampling methods, DNA extraction methods, downstream bioinformatics analysis and obviously contaminations [33]. The present work aims to simultaneously compare the potential effects of the method of placental removal, area sampled and analysis methods on the isolation of bacteria in the *in utero* environment. Several areas of the *in utero* environment (umbilical cord and fetal membranes), together with inner area of the placenta (chorionic villi) were sampled after cesarean section with or without an Alexis^®^ wound retractor device or after vaginal delivery. Several means of microbiota analysis methods were tested. Aerobic and anaerobic bacterial cultures were performed to detect dominant and subdominant cultivable bacteria. Molecular techniques (qPCR and whole genome shotgun metagenomics) were used to assess the presence of dominant cultivable and uncultivable bacteria.

## Materials and methods

### Cohort—Sampling

This project was approved by an ethical committee of the “pôle recherche clinique” of the “ITMO santé publique” from INSERM (n°CPP 2015-mai-13909). Thirty-eight women were recruited from three maternities (Port Royal, Antoine Béclère, and Sainte Félicité). Patients were included after ≥ 37 weeks of gestation. Exclusion criteria included HIV infection, hepatitis, chromosomal abnormalities, fetal malformation, fetal death, pre-eclampsia, premature rupture of membranes, chorioamnionitis. Informed consent was obtained from all the subjects and methods were carried out in accordance with the relevant guidelines and regulations. Placentas were collected following vaginal delivery (VD; n = 9) or cesarean section with (CSA; n = 23) or without (CS; n = 6) an Alexis^®^ wound retractor device. CS and CSA groups received cefazolin (2 mg) prior to delivery and all women had intact membranes. Rupture of membranes prior to delivery in VD group did not exceed 10 h and women did not receive antibiotics. Once collected, placenta was placed in a sterile bucket, kept at 4°C for < 6 h before being dissected. Chorionic villi, umbilical cords and fetal membranes were sampled and transferred into dry cryotubes or cryotubes with 1 mL of Brain Heart Infusion broth (Oxoid, United Kingdom) + 30% glycerol as a cryoprotectant, known to prevent significant changes in the composition of microbiota (BHIG). Samples were then stored at -80°C.

### Bacterial culture

Samples in cryotubes containing BHIG were thawed on ice and transferred under a laminar flow hood. Tissue samples (mean weight for fetal membranes 0.6 ± 0.2 g; umbilical cords 1.0 ± 0.5 g; chorionic villi 0.7 ± 0.3 g) were cut and transferred with the BHIG in a sterile tube. Samples were kept on ice and submitted to 4 x 15 s shredding sessions with an ultra-turrax^®^ T25 (Janke & Kunkel IKA; Laboratechnik; Wasserburg; Germany) disperser tool. Then, 100 μL of the homogenate were plated on two Columbia agar base supplemented with blood (5%; v/v; Oxoid) and cysteine (160 mg/L) media and on two Chocolate agar PolyViteX media (Biomérieux; Marcy l’Etoile; France), and incubated at 37°C either 3 days in aerobic conditions or 5 days in anaerobic conditions (N_2_:H_2_:CO_2_; 80%:10%:10%). Control tubes with the same BHIG batch without tissue followed all the culture process and were plated and incubated in a same manner. We performed 38 controls for each media and incubation condition. Each sample set (chorionic villi, fetal membranes and umbilical cord) from one woman was associated with one negative control. Each type of colony was counted before being identified and conserved in BHIG (1 mL) at -80°C. Routine laboratory methods, *i*. *e*. macroscopic and microscopic observations, and standard biochemical determinations were used for bacterial identification. When necessary, genus and/or species were Sanger sequenced targeting the 16S RNA encoding gene using primers SD008 (5'_AGA GTT TGA TCC TGG CTC AG_3') and SD1492 (5'_ACG GCT ACC TTG TTA CGA CTT_3'.

### DNA extraction

DNA was extracted from placental samples following the recommendation from the Human microbiome project (HMP) with the use of the Power Soil^®^ DNA isolation kit (Qiagen; Hilden; Germany). Tissue in dry tube was thawed on ice and cut under laminar flow hood. Then, tissue (0.25 g) was transferred into the PowerSoil^®^ beads tube, vortexed for 1 min and incubated at 65°C for 10 min and 95°C for 10 min. Lysis step was performed using a Tissue Lyzer^®^ (Qiagen; 5 min at 25 laps/s). DNA concentration was checked using a Nanodrop^™^ (Thermo Scientific^™^; Waltham; Massachussets; USA) and tubes were kept at -20°C. One control (a PowerBead tube^®^ without tissue that followed all the steps of the protocol) was performed at each DNA extraction experiment which represents 16 blanks of extraction.

### qPCR analysis

Inhibitors presence was first analyzed using a Taqman^™^ exogenous internal positive control (IPC^®^; Applied Biosystems^®^) following the recommendations of the supplier.

Then, analysis of bacterial DNA was performed by qPCR targeting V8-V9 region of the gene encoding for 16S rRNA, using Power SYBR^™^ Green PCR Master Mix (Applied Biosystems; Foster City; California; USA), F_PROK1369 (5’_CGGTGAATACGTTCCCGG_3’) and R_PROK1492 (5’_TACGGCTACCTTGTTACGACTT_3’) primers. Mix for 1 reaction was composed of SYBR^™^ Green PCR Master Mix (1X), F_PROK1369 (0.3 μM), R_PROK1369 (0.3 μM), DNA template (6 μL; 1:5 diluted) and completed with sterile MQ^®^ water to 20 μL. The following program was used: 95°C 10 min, 40x (95°C 30 s, 60°C 30 s), 95°C 15 s, 60°C 15 s, 95°C 15 s. Two negative controls were added in each qPCR run: a DNA extraction without tissue (blank) and a sample of MQ^®^ sterile water. Results were analyzed with CFX manager 3.1 software.

### qPCR threshold detection

qPCR threshold detection was determined by contaminating the chorionic villi extracted DNA with several quantities of *E*. *coli* TOP10 DNA (5 to 5 x 10^−6^ ng) which was extracted using phenol-chloroform based manual extraction protocol and was added at the end of the PowerSoil^®^ DNA isolation protocol. Then, bacterial DNA presence was analyzed by qPCR as described.

### Shotgun metagenomics

DNA samples and negative controls were used to make fragment libraries using the TruSeq Library Kit (Illumina, San Diego, CA). Resulting libraries were sequenced on an Illumina HiSeq2500 to generate single-end fragment reads of 65 bp. Single-read, as opposed to paired-ends, sequencing was performed based on the intended analysis which only required unique matching to existing databases. Assembly of the data was not considered due to extremely low bacterial biomass and previously-reported placental species are well-represented in the database. Sequence data were filtered and demultiplexed using the Illumina-supplied program bcl2fastq2 (v2.20) with default parameters.

Filtering of Eukaryotic reads (Human) was performed with Bowtie2. A database built from the human genome hg38 ‘no_alt_plus’ Bowtie2 index files, available at NCBI (ftp.ncbi.nlm.nih.gov), was used as a reference [34]. Bowtie2 was run with the ‘—local’ and set to output unaligned reads. Raw data, with human sequences removed were deposited in the European Nucleotide Archive (ENA) and can be retrieved using the study accession number #PRJEB37487. Identification of the resulting unaligned, filtered, reads was performed with KrakenUniq [35]. KrakenUniq was run with default parameters and the resulting report files were visualized using Pavian [36].

The latest RefSeq and ‘nt’ reference databases for KrakenUniq were obtained from ftp://ftp.ccb.jhu.edu/pub/software/krakenuniq/Databases/. Earlier versions of the RefSeq catalog were reconstructed following the procedure described previously [37]. Resulting FASTA files were used to generate KrakenUniq databases following instructions provided in the Kraken manual (http://ccb.jhu.edu/software/kraken/MANUAL.html) and at https://github.com/fbreitwieser/krakenuniq.

### Statistical analysis

Statistical tests were realized using Kruskal-Wallis test to compare 3 populations or Mann-Whitney test to compare 2 populations. All statistical analyses were performed with GraphPad software (Prism). Differences were considered significant when the P value was < 0.05.

## Results

### Description of the cohort

A total of 38 women giving birth at full term without any pregnancy complications were recruited from the three maternities (Port Royal n = 17, Sainte Félicité n = 9, and Antoine Béclère Hospital maternity n = 12, Table 1). No significant differences in age or length of gestation were found between the 3 centers or the 3 collection methods. For technical reasons, we did not obtain all the samples from all women and we did not perform all the techniques on all samples. Thus, bacterial presence in 34 fetal membranes, umbilical cords and chorionic villi was studied using bacterial culture and qPCR. Among these 34 inclusions, 3 chorionic villi samples were also analyzed by metagenomics. Moreover, 4 supplementary chorionic villi samples were analyzed by culture and metagenomics (Table 1).

**Table 1:** Main characteristics of the cohort.

Group	Delivery mode	Centre	n	Median age	Median WG	Patient number	Culture	qPCR	Metagenomics
CSA	Cesarean section + Alexis	1	17	38 ± 4	39 ± 0.8	P1, P3, P4, P7 to P13	X	X	
						P2, P5, P6	X	X	X
						P35 to P38	X		X
		2	3	37 ± 6	39.1 ± 1.2	P14 to P16	X	X	
		3	3	36 ± 5	39.1 ± 0.7	P17 to P19	X	X	
CS	Cesarean section	2	3	36 ± 7	39.3 ± 0.3	P20 to P22	X	X	
		3	3	38 ± 4	39.3 ± 0.1	P23 to P25	X	X	
VD	Vaginal delivery	2	6	35 ± 5	39.1 ± 1.3	P26 to P30, P34	X	X	
		3	3	32 ± 3	39.9 ± 0.5	P31 to P33	X	X	

No significant differences were found regarding age or week of gestation between all groups using Kruskall-Walis test. Centre 1: Port Royal maternity; Centre 2: Antoine Béclère Hospital maternity; Centre 3: Sainte Félicité maternity; n: number of patients; WG: weeks of gestation.

### Aerobic and anaerobic cultures

Samples were studied using bacterial culture to assess the presence of potential viable bacteria in the fetal membranes, umbilical cord and chorionic villi in parallel with negative controls.

In total, 136 control Petri dishes were inoculated with 100 μL of BHIG and 10 different bacterial species (with less than 10 CFU/ Petri dish) were isolated as contaminants on 14 plates. Identified contaminants were: (CN)-staphylococci, *Kocuria rhizophila*, *Nocardiopsis synnemataformans*, *Cutibacterium acnes*, *Bacillus firmus*, *Micrococcus spp*., and *Moraxella osloensis*. In our study, we tested two methods to include negative controls in the analysis: *i) r*emoving bacterial species from a sample if we found this species in the corresponding control (which corresponds to the results showed in S1 Table) or *ii)* removing bacterial species found in a negative control in all samples in which we found this species. For the first analysis showed in S1 Table, we found 70% (CSA), 33% (CS) and 56% (VD) of positive chorionic villi samples; 79% (CSA), 50% (CS) and 100% (VD) of positive umbilical cord samples and 68% (CSA), 17% (CS) and 100% (VD) of positive fetal membranes samples. With the second analysis, the number of positive samples with bacterial species dramatically dropped. Precisely, in chorionic villi, we found 48% (CSA), 17% (CS) and 44% (VD) of positive samples. In umbilical cords, we found 48% (CSA), 0% (CS) and 100% (VD) of positive samples and in fetal membranes, we found 37% (CSA), 17% (CS) and 100% (VD) of positive samples.

**Fig 1:**
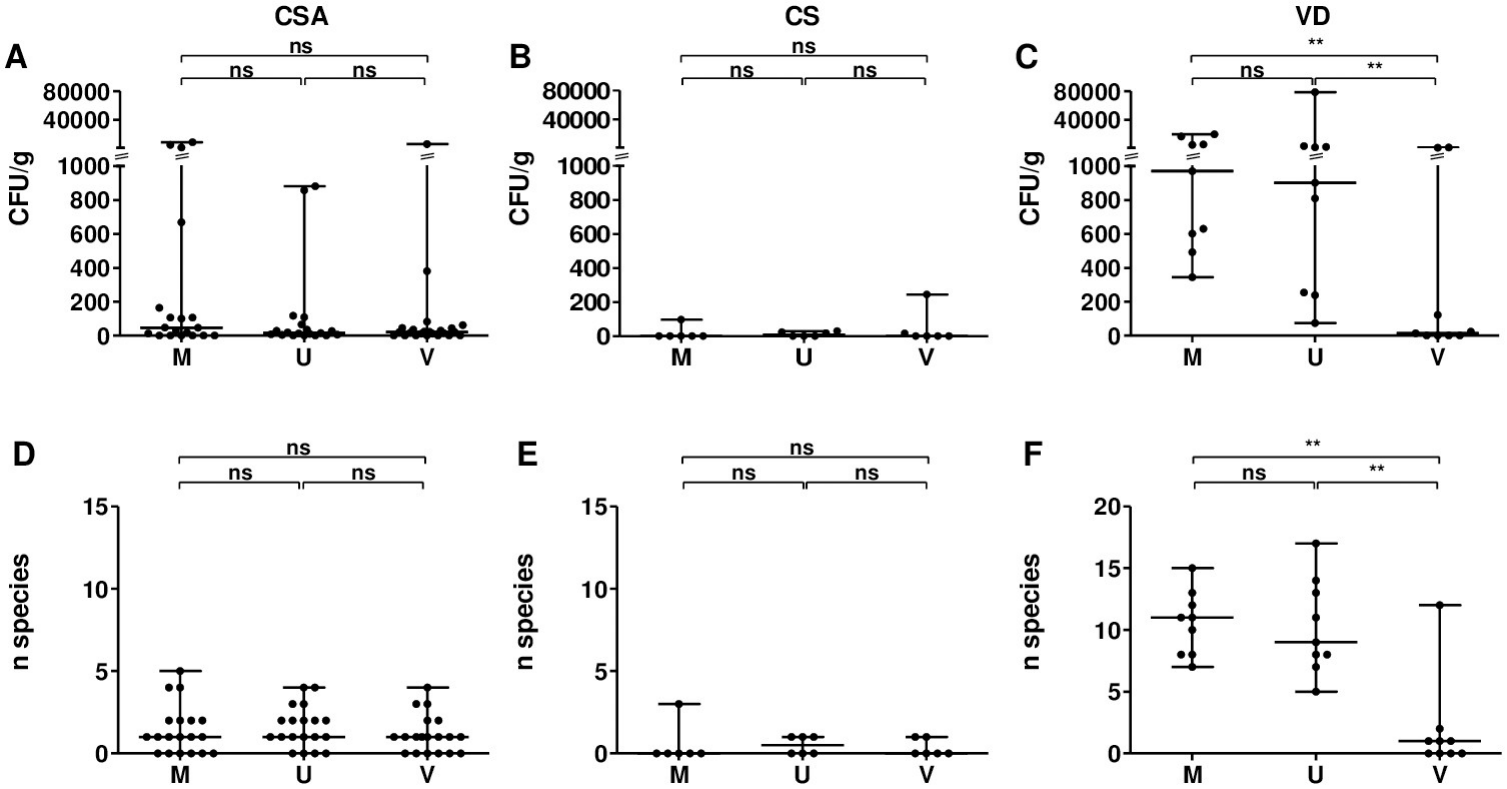
Quantity of bacteria (A-C) and number of different species (D-F) found in fetal membranes, umbilical cords and chorionic villi collected after cesarean section using an Alexis^®^ (CSA group; A, D), cesarean section (CS group; B, E) or vaginal delivery (VD group; C, F). CFU: colony forming unit; CSA: cesarean section using an Alexis^®^; CS: cesarean section; VD: vaginal delivery; M: fetal membranes; U: umbilical cord; V: chorionic villi. ns: non-significant; *: P < 0.05; **: P < 0.01.

In the CSA (n = 23) and CS groups (n = 6), viable bacteria were occasionally found generally in scarce quantities independently of the area sampled (Fig 1A and 1B). Both groups yielded a low number of species (Fig 1D and 1E). A relatively elevated quantity of bacteria (≥ 8.0 x 10^2^ CFU/g of tissue) was observed for 3 fetal membranes (P1, P2 and P9), 2 umbilical cords (P2 and P9) and 1 chorionic villi (P19) all from the CSA group, whereas two sample sets from both groups (P12 and P20) were free of cultivable bacteria in all areas. Chorionic villi from P37 (which was the unique area we analyzed for this patient) was also free of bacteria. Otherwise, we observed a more pronounced bacterial detection in the CSA group than in the CS group which is significant only for the fetal membranes (P = 0.049). In the VD group (n = 9), bacteria were isolated from all fetal membranes and umbilical cords with significantly higher quantities of bacteria and species than found in chorionic villi (Fig 1C and 1F). Medians of 9.7 x 10^2^ and 9.0 x 10^2^ CFU/g of tissue and medians of 11 and 9 different species were found in fetal membranes and umbilical cords, respectively. However, as in CSA and CS groups, viable bacteria were observed in only five chorionic villi and were in low abundance (median of 14 CFU/g of tissue), associated with a small number of species (median of 1 species per sample). Two samples set (from P26 and P31) showed a higher quantity of bacteria (each 1.0 x 10^3^ CFU/g of tissue) than others but were characterized by longer time interval between membranes rupture and freezing of the samples (both > 9h).

**Fig 2:**
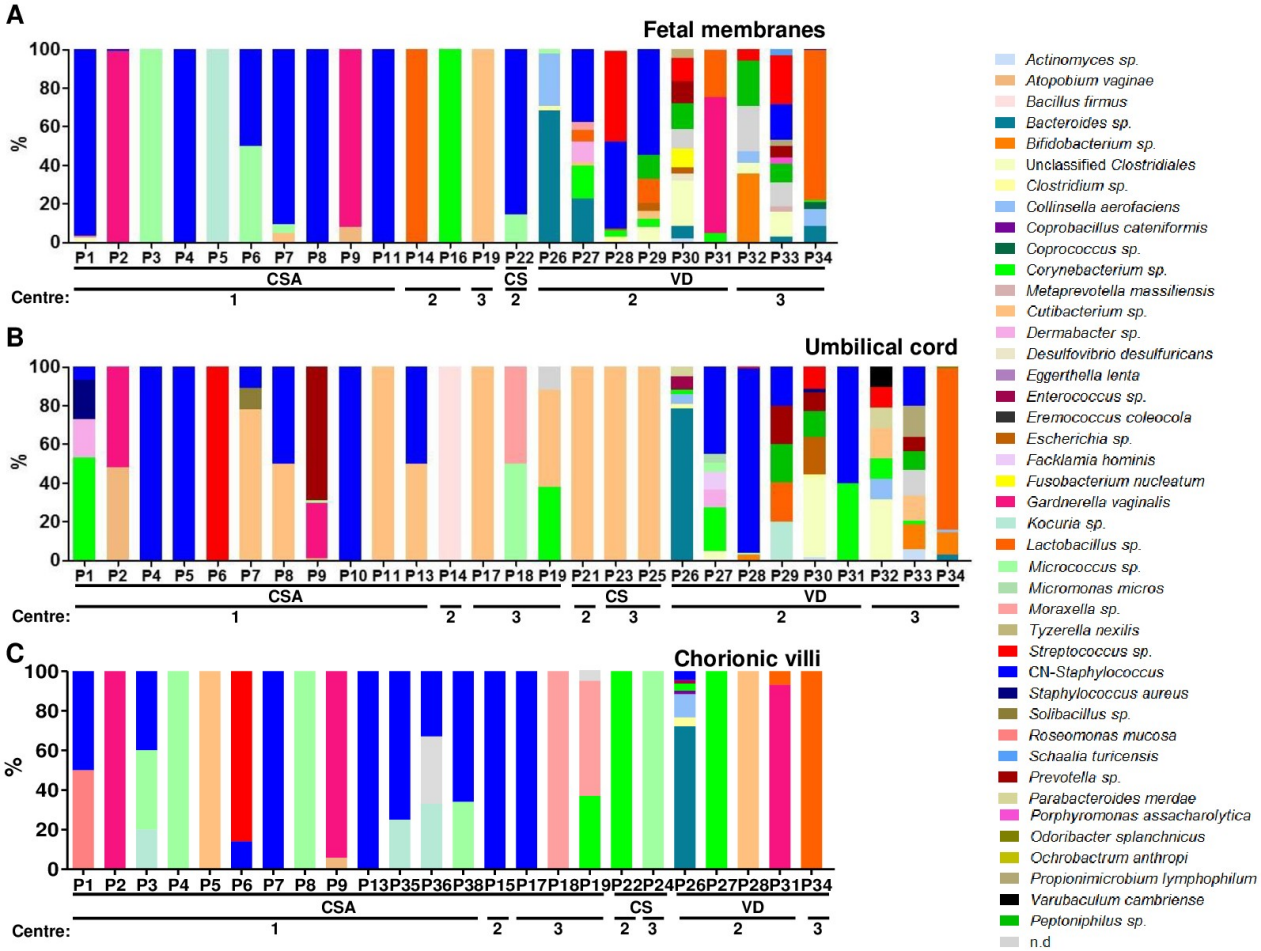
Prevalence (%) of each bacterial species/genus in fetal membranes (A), umbilical cords (B) and chorionic villi (C). Samples that did not harbor any bacteria were removed. The higher diversity was observed in external samples (fetal membranes and umbilical cords) coming from vaginal delivery. CSA: cesarean section with Alexis^®^; CS: cesarean section; VD: vaginal delivery; Centre 1: Port Royal maternity; Centre 2: Antoine Béclère Hospital maternity; Centre 3: Sainte Félicité maternity.

CSA and CS groups isolated bacteria were predominantly coagulase negative (CN)-staphylococci, *Micrococcus sp*., and *Cutibacterium acnes* which were also on negative controls (Fig 2). P1 *in utero* environment was characterized by a high quantity of (CN)-staphylococci (1.4 x 10^3^ CFU/g of tissue) in fetal membranes (Fig 2A). Elevated quantities of *Gardnerella vaginalis* in P2 and P9 fetal membranes (5.1 x10^3^ and 8.0 x 10^3^ CFU/g of tissue respectively) were observed (Fig 2A and 2B). This species was also found in umbilical cord and in chorionic villi of both samples but in lower quantity (< 5.0 x 10^2^ CFU/g of tissue). Finally, elevated quantities of *Moraxella osloensis* and *Corynebacterium accolens* were observed in chorionic villi from P19 (3.6 x 10^3^ and 2.3 x 10^3^ CFU/g of respectively; Fig 2C). In the VD group, a higher diversity of bacteria was found compared to CSA and CS groups both in fetal membranes and umbilical cords. Predominant genera found were usually vaginal or fecal microbiota associated bacteria (Fig 2A–2C). Chorionic villi from P26 and P31 which were characterized by a long time between rupture of membranes and freezing of the samples were found to harbor elevated quantities of *Bacteroides sp*. and *Gardnerella vaginalis* (1.0 x 10^3^ and 8.5 x 10^2^ CFU/g of tissue respectively; Fig 2C).

### 16S targeted qPCR

We investigated bacterial DNA presence using qPCR. First, we performed quenching control qPCR reactions using a Taqman^™^ exogenous internal positive control kit (IPC kit). We found that DNA detection improved (lower Ct) at higher dilutions, indicating the presence of inhibitors in the extracted DNA. Dilutions higher than 1:5 had little additional effect on Ct and we used this dilution for further qPCR detection of bacterial DNA in samples (S1 Fig). The detection threshold of bacterial DNA was determined in chorionic villi by adding a known quantity of *Escherichia coli* TOP 10 genomic DNA (from 5 to 5 x 10^−6^ ng). A threshold of 5 pg of DNA was determined as DNA was detected earlier in the negative control at concentrations of ≤ 5 pg of *E*. *coli* DNA.

**Fig 3:**
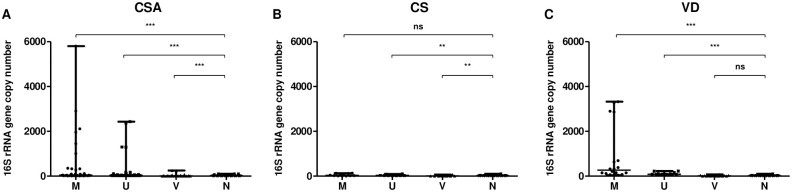
16S rRNA gene copy number in fetal membranes, umbilical cords, chorionic villi and extraction blanks collected after C-section with (A) or without (B) an Alexis^®^ or after vaginal delivery (C). CSA: cesarean section using an Alexis^®^; CS: cesarean section; VD: vaginal delivery; M: fetal membranes; U: umbilical cord; V: chorionic villi. ns: non-significant; **: P < 0.01; ***: P < 0.0001.

Then, qPCR was performed on the extracted DNA from chorionic villi, umbilical cords and fetal membranes. Each reaction included extraction blanks performed during DNA extraction. Extraction blanks harbored a median of 18 16S rDNA gene copy number. In CSA group, the 16S rDNA gene copy number was significantly higher in fetal membranes (median = 36) and umbilical cords (median = 29) extracted DNA compared to extraction blanks (Fig 3A). However, it was significantly lower in chorionic villi extracted DNA (median = 5). In CS group, 16S rDNA gene copy number was similar in fetal membranes (median = 31) and significantly lower in umbilical cords (median = 30) and chorionic villi (median = 6) extracted DNA compared to extraction blanks (Fig 3B). In VD group, 16S rDNA gene copy number was similar in chorionic villi extracted DNA (median = 12) compared to extraction blanks whereas it was significantly higher in fetal membranes (median = 266) and umbilical cords (median = 82) extracted DNA (Fig 3C).

### Whole genome shotgun metagenomics

DNA from 7 chorionic villi samples (P2, P5, P6, P35, P36, P37 and P38) was analyzed by high-throughput metagenomics analysis. Three reagents extraction kit controls (blanks) were analyzed in parallel. Using an Illumina HiSeq 2500, we generated an average of 1.9 x 10^7^ fragment reads for biological samples and an average of 4.9 x 10^5^ fragment reads for control samples. The scarcity of bacterial reads was again evident in these samples. We first filtered sequence reads by mapping against the human genome (hg38). Chorionic villi samples were found to contain over 99% of human derived reads and post-filtering data averaged 1.1 x 10^5^ remaining reads per sample. Negative controls showed highly variable human DNA content ranging from 50% to 74%, and an average of 1.7 x 10^5^ reads remaining after filtering.

Identification of remaining reads was performed with KrakenUniq against both the latest RefSeq and full nucleotide ‘nt’ microbial databases. Interestingly, we were only able to identify an average of 6.0% (6,153) of reads in placental extract samples using the bacterial ‘nt’ database from NCBI. *Ralstonia solanacearum* dominated identification with 2,242 average reads representing 36% of all identified reads in chorionic villi. This was similar to the negative controls where *Ralstonia* was the top hit with 45,700 average reads representing 58% of average identified reads in extraction blanks (S2A Fig).

We complemented the above analysis by also mapping data to the RefSeq database, which includes representative organisms across both eukaryotic and prokaryotic kingdoms as well as viral sequences. RefSeq also includes a range of artificial sequences associated with cloning and library preparation kits. When comparing sequence reads against RefSeq, we found 23% of reads from placental samples were of human origin, whereas < 0.5% were identified as human in negative controls. Since KrakenUniq uses k-mer analysis, as opposed to direct sequence alignment for Bowtie, it is expected that some additional human reads might be mapped. Empty vectors or other exclusively artificial sequences accounted for between 0.7% and 5.6% of reads in placental samples and over 42% of reads in control samples. In agreement with culture and qPCR techniques, very few bacterial species were found in placental samples. *Escherichia*, *Xanthomonas*, *Pseudomonas* or *Staphylococcus* were the top genera depending on which database was used and these genera were found in all tissue samples and negative controls (S2B and S2C Fig).

In comparing results obtained from both ‘nt’ and RefSeq databases, we noticed differences beyond what would be expected from their respective compositions. We observed that *R*. *solanacearum* is particularly notable in its absence among the top hits using RefSeq, even though it was the most prevalent organism identified when comparing against the ‘nt’ database. We also noted that while RefSeq proved quite useful in identifying potential artificial sequences, it also yielded a generally lower species diversity prediction. Based on these results we wished to investigate to what degree database selection and identification of different sources of contamination could influence results.

**Fig 4:**
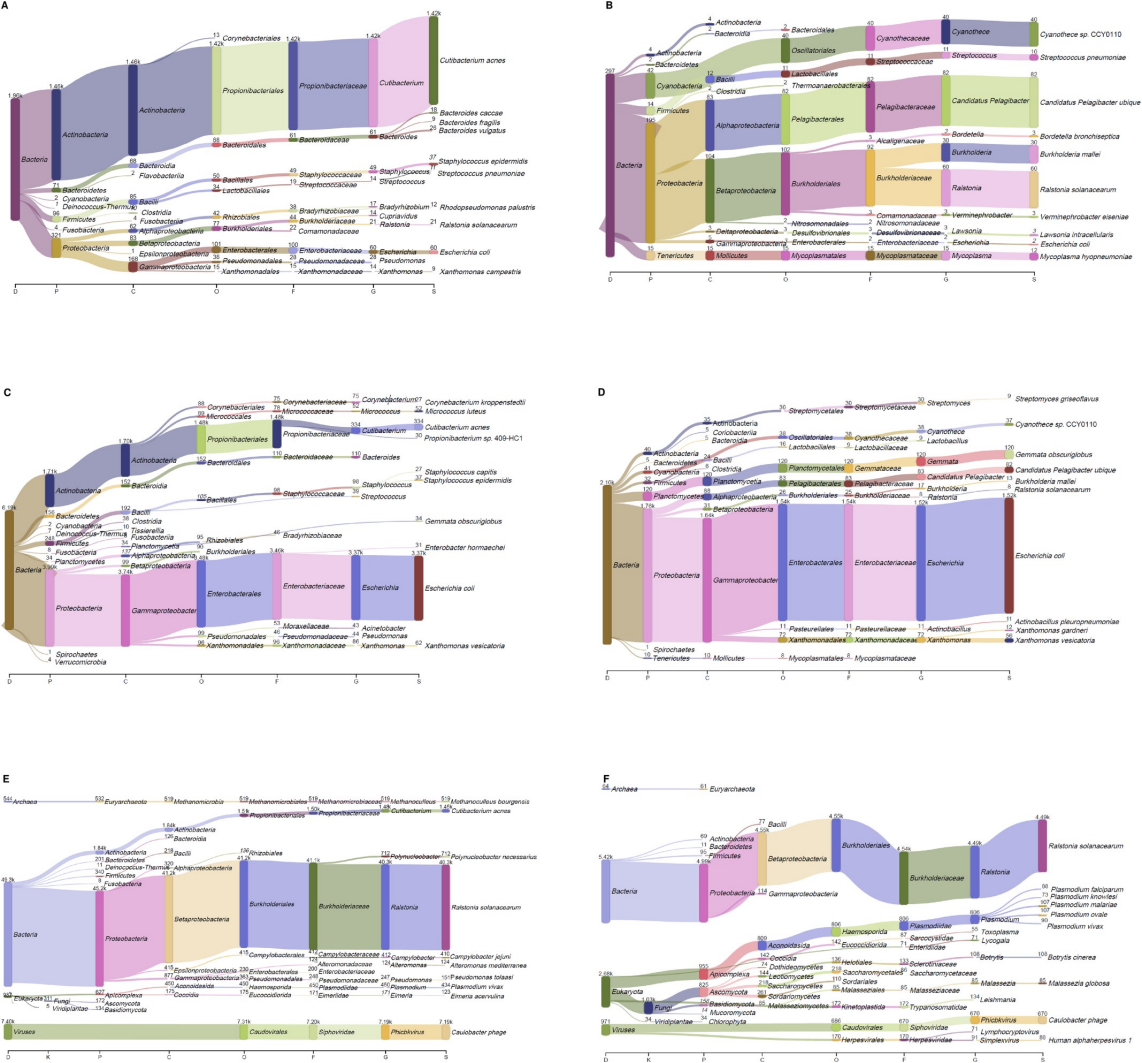
Example of microbial composition in negative control (A, C, E) or chorionic villi (B, D, F) using different databases for analysis with KrakenUniq. DNA was found in both negative control and chorionic villi and was highly variable depending on the database. Databases used: RefSeq v24 (A, B); RefSeq v48 (C, D); NCBi ‘nt’ (E, F).

We further investigated the impact of database by reconstructing previous versions of RefSeq, from v12 (July 2005) until v70 (July 2015). Examination of control samples revealed that a significant number of bacterial species could be erroneously interpreted as being present in placental extracts if not properly subtracted. Moreover, depending on the version of RefSeq used, different results were obtained. For example, in P2 associated negative control, the major representative was either *C*. *acnes* or *E*. *coli* (Fig 4A and 4C). Analysis using the ‘nt’ database retrieved a similar number of reads (1,460) for *C*. *acnes* but also found 27 times as many hits for *R*. *solanacearum* (Fig 4E). Chorionic villi from P2 were analyzed in the same manner. A significant number of reads are unassigned in versions of RefSeq prior to v48, where the dominant species identified is *E*. *coli* (Fig 4B and 4D). *E*. *coli* is completely absent in analysis carried out using the ‘nt’ database, where the predominant predicted species is again *R*. *solanacearum* (Fig 4F).

## Discussion

In the context of the debate on the existence of a “placental microbiota”, our study assessing a range of placental removal methods, of sampled areas and of analysis methods allows us to conclude that we did not find convincing evidence for the existence of a resident, specific and functional microbiota. Moreover, we clearly demonstrated that isolation of bacteria or bacterial DNA in the *in utero* environment was associated with exposure of the samples to contamination during placental delivery. Indeed, one of the most important conclusion is that the use of appropriate negative controls in such samples with very low biomass are an absolute necessity in order to reach valid conclusions. We also showed that sequencing data generated with metagenomics could be misinterpreted; indicating that database choice has to be checked.

Although more than 80% of environmental bacteria are not readily cultivable, bacterial culture can provide information about community microorganisms present in low abundance and, importantly, about their viability and potential metabolic activity [38, 39]. Using bacterial culture, we showed that the chorionic villi representing the inner part of the placenta were rarely colonized. Moreover, bacteria identified were usually associated with cutaneous microbiota, fecal, vaginal or environmental bacteria. For example, we found bacteria in 5 chorionic villi from VD group with a number of different species generally low (1 to 3 species except for P26). For P26, we found *Bacteroides sp*., *Clostridium innocuum*, *Collinsella aerofaciens*, *Enterococcus durans*, *Corynebacterium sp*., CN-staphylococci and *Coprobacillus cateniformis*. This composition strongly suggests a fecal contamination. For other samples, we found *Corynebacterium tuberculostearicum*, *Cutibacterium acnes*, two species suggesting a cutaneous contamination and *Lactobacillus crispatus*, *Lactobacillus gasseri* and *Gardnerella vaginalis*, 3 species suggesting a vaginal contamination. Taken together, these observations led us to suggest an external bacterial contamination during the placental collection for these samples. Similar results were observed for external sampling areas such as umbilical cord and fetal membranes collected after cesarean section. However, significantly higher bacterial quantity and diversity were systemically found in fetal membranes and umbilical cords collected after vaginal delivery. Three chorionic villi samples showed a higher quantity of cultivable bacteria of which two experienced extended times between rupture of membranes and freezing of the samples, which was identified as an important factor contributing to higher bacterial quantities detected by qPCR [11]. We also found a more pronounced bacterial detection with the use of an Alexis. This difference is significant when comparing fetal membranes but was not when comparing chorionic villi or umbilical cords. We hypothesized that the extra handling to set up the wound retractor device could be a source of contamination of the fetal membranes. Taken together, these data suggest that the isolation of cultivable bacteria in the *in utero* environment is strongly associated with exposition to contaminants during placental delivery. Previously, studies that employed broad culture conditions, including aerobic and anaerobic growth, showed between 0 and 56% of positive samples [5, 14, 25, 28, 40]. In these studies, *Cutibacterium sp*., (CN)-staphylococci, *Bacillus sp*., *Corynebacterium sp*. *Brevibacterium sp*., *Lactobacillus sp*. were occasionally found in the placenta. As in our study, these isolates were considered as contaminants. Differences in bacterial isolation frequency or bacterial quantity between samples coming either from vaginal delivery or cesarean section was not described in most of molecular studies [8, 13, 21] but had already been observed in two studies using culture techniques [5, 40]. Interestingly, amnion-chorion portions or transmural parenchyma (which can contact the exterior of the placenta) were sampled in these studies. Along with our results with inner and external areas of the placenta, it clearly showed the importance of delivery mode and sampling method as factor which can cause misinterpretation.

Here, qPCR targeting the V8-V9 region of the 16S rRNA encoding gene did not allow detection of bacterial DNA in chorionic villi samples regardless of the delivery method despite viable bacteria being observed in some samples by bacterial culture. Indeed, chorionic villi extracted DNA was characterized by a lower quantity of 16S gene copy number compared to controls. The sensitivity of our analysis was estimated at 5 pg of *E*. *coli* DNA, which is in the same order as a previous study [25] and corresponds to approximately 4.0 x 10^3^ CFU/g of chorionic villi. However, significantly higher 16S gene copy number was detected in fetal membranes and in umbilical cords collected after cesarean section using an Alexis^®^ or after vaginal delivery suggesting that DNA from placental delivery associated contaminant was detected. Interestingly, such differences were not found in samples coming from classical cesarean section but the small sized sample set can cause a lack of representatively. Other studies detected bacterial DNA from 0 to 47% in chorionic villi samples by qPCR [11, 13, 25–28]. Results variability can be explained by the differences in area sampled and molecular methods used. Indeed, targeting V1-V2 or V2 region of the 16S rRNA encoding gene, no bacterial DNA was detected no matter which placenta area was sampled [25–28]. Targeting V5-V7 region, bacterial DNA was detected in 47 and 68% of chorionic villi and fetal membranes respectively [11]. In this study, the higher bacterial quantity observed was usually associated with low diversity suggesting a potential contamination. Moreover, higher quantity was observed when time before freezing samples was long. The V4 region of the 16S rRNA encoding gene was previously recommended to study “placental microbiota” using qPCR [13]. Here, qPCR targeting V4 region was performed according to the previously published protocol [13] but it did not reach sufficient efficiency (< 90%). However, the predominant DNA detected using V4 region was shown to belong to *Ralstonia* genus, a bacterial genus generally found in soil with some species described as plant pathogen, such as *R*. *solanacearum* (the species we found by metagenomics). This genus is commonly associated with contaminations. Moreover, a recent published analysis showed that bacterial DNA was undetectable using V4 region targeted PCR in 28 samples coming from 4 placentas collected after cesarean section [25]. Here, we showed that bacterial DNA was not detected inside chorionic villi targeting a previously untargeted region (V8-V9) of the 16S rRNA encoding gene.

In our metagenomic analyses, the identification of a predominance of *R*. *solanacearum* reads in both placental and control samples appears to be consistent with previously reported laboratory contaminations [24–28, 41]. This type of contamination can have an outsized effect on the analysis of low-biomass environments. Metagenomics also allowed us to identify common cutaneous contaminants found also in operating rooms including both *Corynebacterium* and *Staphylococcus* which were also isolated using bacterial culture [42]. In each case, these species were found both in negative controls and placental samples as previously found [24, 28]. Thus, metagenomics confirmed results obtained from chorionic villi analyzed using culture and qPCR experiments while also raising important questions concerning the effect of how high-throughput sequencing data from low-biomass environments are analyzed. Our investigation sought both to explore the possibility of a “placental microbiota” as well as the potential factors, including contaminations, which have led other groups to report on such a microbial community. We explored both samples and negative controls without any additional filtering beyond the removal of human-derived reads. Our results confirm the results from other studies that demonstrate that identification and removal of contaminating sequences remains a challenge, especially for environments with unknown composition [43].

There is now mounting evidence that contamination from the environment can substantially affect the results of high-throughput sequencing analysis on low-input samples. Laboratory reagents, kits or the sequencing instruments themselves are potential sources of contaminations [41, 44]. Researchers would presumably expect a small minority of reads from low-input samples to be microbial in nature. Indeed, over 99% of experimental samples reads were found to be human, leaving an average of only 106K out of 20 million reads. In order to identify the maximum number of potential organisms from the remaining reads, we chose two comprehensive databases; the microbial nucleotide ‘nt’ database and the RefSeq (v86) database. A comparison of results revealed major differences. A significant number of control sample reads matched *C*. *acnes* with both databases, however the ‘nt’ database also allowed for the identification of *R*. *solanacearum* as the major constituent. Given that *Ralstonia* species were the dominant organisms recovered in both control and experimental samples, and that this organism has been associated with contamination, we found its ‘nt’ database-specific detection to be surprising. It should also be noted that these observed differences, while quite remarkable in low-input samples, nevertheless only represent < 0.05% of the reads.

To investigate what specific differences could be database dependent in these low-input samples, we used archived compositions of previous RefSeq versions. We reconstructed and used RefSeq versions, for KrakenUniq, spanning a decade since the introduction of next generation sequencers, 2005–2015. As expected, the percentage of identified reads increased with later, and presumably more complete, versions of the database. What was quite interesting was the highly variable taxonomic assignment made depending on the database version. The inclusion and filtering of synthetic reads (cloning vectors or sequencing kits for example) along with human sequences greatly reduced the number of reads tagged as microbial in the analysis. This suggests that the misidentification of these ubiquitous DNA fragments could contribute to the erroneous presumption of a stable bacterial community in some samples. A final point concerns the size and complexity of such databases used to perform the analysis. As the amount of available data continues to increase through successive versions of both RefSeq and the ‘nt’ databases, greater and greater computational power is necessary to setup and to perform analyses. We noted that v12 of RefSeq, with 1 x 10^6^ entries, was generated with 5.5 x 10^9^ unique 31-mers. Generating v70, with 1.3 x 10^8^ entries (130x of v12) resulted in 2.7 x 10^10^ 31-mers (only 5.5x more than for v12). Indeed, the optimal number of k-mers could not be loaded into memory (512 Gb) for any RefSeq versions after v59, and so the final number of k-mers, and resulting resolution, were less than optimal and resulted in a commensurate loss in taxonomic identification. RefSeq versions later than v80 have very high memory requirements and require several days, to weeks, to build on state-of-the-art clusters. Options to overcome these limitations include increasing k-mer length, restricting k-mer numbers or reducing the size of the database. All options will tend to limit or bias detection and make comparisons with previous studies difficult. Taken together with the variability observed between databases and database versions, these observations help explain the inconsistent results obtained in studies examining the “placental microbiota”. Since few laboratories will be able to construct their own databases for comprehensive analysis, one must remain vigilant to understand the composition and characteristics of pre-compiled databases for analysis.

Data from previous molecular biology based studies showed the predominant presence of *E*. *coli* [8]. Here, *E*. *coli* DNA was found to be predominant in negative controls and in chorionic villi when sequencing results were analyzed with the v48 of RefSeq. However, abundance of *E*. *coli* DNA dramatically dropped when another version of RefSeq or ‘nt’ databases were used, showing the importance of database choice to analyze metagenomic data. Recently, *E*. *coli* DNA was evidenced in chorionic villi samples [24]. However, supplementary analysis showed that this DNA found in several chorionic villi samples belonged to one unique strain. Thus, *E*. *coli* DNA was considered as contaminant. Moreover, the cultivable *E*. *coli* species was not found in chorionic villi samples using culture methods, as reported before [1, 5, 14, 25, 28, 40]. It was occasionally found only in external areas of the placenta coming from VD group. All these data point to the absence of *E*. *coli* and *E*. *coli* DNA inside the placenta.

To conclude, this is, to our knowledge, the first study that analyzed bacterial presence inside the placenta (chorionic villi) and in its environment (umbilical cord and fetal membranes) by culture and molecular techniques, using samples collected after several delivery methods and corresponding controls. We found that no significant quantities of viable bacteria or bacterial DNA were detectable in the *in utero* environment samples collected after cesarean section. To be able to isolate a significant quantity of viable bacteria or bacterial DNA, samples had to be in contact with the exterior of the placenta and be collected after vaginal delivery highlighting the contamination risks. Moreover, higly sensitive metagenomics data analysis can often lack specificity and cause misinterpretation of results depending on the database choice and negative controls used.

As absence of evidence of bacterial presence in placenta cannot be considered an absolute proof, this work will continue to add to the increasing number of publications which together conclude that the placenta does not harbor a microbiota.

## Supporting information

S1 Table Bacterial species/genus found in each sample. Samples from P12, P20 and P37 were removed due to the absence of bacteria. A higher number of different species was observed in the samples from the VD group and more specifically in fetal membranes and umbilical cords when compared to CSA and CS groups. Bacterial species found in a control were removed from the corresponding sample. CN: coagulase negative; -: no bacteria found; n. d.: bacterial identification not determined; ?: experiment not performed.
(DOCX)

S1 Fig Threshold cycle showing detection of control DNA with the IPC kit using several dilutions of extracted DNA from fetal membranes, umbilical cord and chorionic villi, to assess the presence of inhibitors. As the dilution factor of extracted DNA increased, the detection of control DNA was earlier indicating presence of inhibitors in extracted DNA from all areas sampled. The 1:5 dilution seemed to eliminate most of inhibitors since it did not drastically vary with higher dilution factors. M: fetal membranes; U: umbilical cord; V: chorionic villi.
(TIF)

S2 Fig Bacterial profiles in negative controls (extraction blanks) and chorionic villi samples using ‘nt’ (A), RefSeq v.48 (B) or RefSeq v.70 (C) for analysis with KrakenUniq. N: negative control; P: placental sample.
(TIF)
