# Supplementary material for: Evidence for contamination as the origin for bacteria found in human placenta rather than a microbiota
Source: PLoS One. 2020 Aug 10;15(8):e0237232. doi: 10.1371/journal.pone.0237232 (PMC7416914; doi:10.1371/journal.pone.0237232)
Supplement: S1 Table — Samples from P12, P20 and P37 were removed due to the absence of bacteria. A higher number of different species was observed in the samples from the VD group and more specifically in fetal membranes and umbilical cords when compared to CSA and CS groups. Bacterial species found in a control were removed from the corresponding sample. CN: coagulase negative; -: no bacteria found; n. d.: bacterial identification not determined; ?: experiment not performed. (DOCX) [file pone.0237232.s001.docx]

**S1 Table**. Bacterial species/genus found in each sample. Samples from P12, P20 and P37 were removed due to the absence of bacteria. A higher number of different species was observed in the samples from the VD group and more specifically in fetal membranes and umbilical cords when compared to CSA and CS groups. Bacterial species found in a control were removed from the corresponding sample. CN: coagulase negative; -: no bacteria found; n. d.: bacterial identification not determined; ?: experiment not performed.

| Patient | Group | Fetal membranes | Umbilical cords | Chorionic villi |
| --- | --- | --- | --- | --- |
| P1 | CSA | CN-staphylococci*,* *Rosemonas mucosa,* *Finegoldia magna* | CN-staphylococci*,* *Staphylococcus aureus,* *Dermabacter vaginalis, Corynebacterium striatum* | CN-staphylococci*,* *Roseomonas mucosa* |
| P2 |  | CN-staphylococci*,* *Gardnerella vaginalis,* *Cutibacterium acnes* | *Gardnerella vaginalis,* *Atopobium vaginae* | *Gardnerella vaginalis* |
| P3 |  | *Micrococcocus luteus* | - | *Micrococcus luteus, Kocuria rhizophila,* CN-staphylococci |
| P4 |  | CN-staphylococci | CN-staphylococci | - |
| P5 |  | *Kocuria rosea* | *Staphylococcus warneri* | *Cutibacterium acnes* |
| P6 |  | *Micrococcus luteus,* CN-staphylococci | *Streptococcus sp.* | CN-staphylococci*,* *Streptococcus sp.* |
| P7 |  | CN-staphylococci*,* *Micrococcus luteus,* *Cutibacterium acnes* | CN-staphylococci*,* *Cutibacterium acnes,* *Solibacillus isronensis* | CN-staphylococci |
| P8 |  | CN-staphylococci | CN-staphylococci*, Cutibacterium acnes* | *Micrococcocus luteus* |
| P9 |  | *Gardnerella vaginalis,* *Atopobium vaginae* | *Gardnerella vaginalis,* *Atopobium vaginae,* *Prevotella bucalis,* *Micrococcus lylae* | *Gardnerella vaginalis,* *Atopobium vaginae* |
| P10 |  | - | *Staphylococcus hominis* | - |
| P11 |  | *Staphylococcus epidermidis* | *Cutibacterium acnes* | - |
| P13 |  | - | *Staphylococcus warneri, Cutibacterium acnes* | *Staphylococcus haemolyticus* |
| P14 |  | *Lactobacillus iners* | *Bacillus firmus* | - |
| P15 |  | - | - | *Staphylococcus epidermidis* |
| P16 |  | *Corynebacterium hansenii* | - | *-* |
| P17 |  | - | *Cutibacterium acnes* | *Staphylococcus capitis* |
| P18 |  | - | *Micrococcus luteus,* *Moraxella osloensis* | *Moraxella osloensis* |
| P19 |  | *Cutibacterium acnes* | *Corynebacterium accolens,* *Cutibacterium acnes* | *Moraxella osloensis,* *Corynebacterium accolens,* *Staphylococccus capitis* |
| P35 |  | *?* | *?* | *Staphylococcus capitis, Staphylococus xylosus, Kocuria marina* |
| P36 |  | *?* | *?* | *Staphylococcus epidermidis, Kocuria varians, n. d.* |
| P38 |  | *?* | *?* | *Staphylococcus auricularis, Staphylococcus saprophyticus, Micrococcus luteus* |
| P21 | CS | - | *Cutibacterium acnes* | - |
| P22 |  | *Micrococcus luteus, CN-*staphylococci | - | *Corynebacterium freneyi* |
| P23 |  | - | *Cutibacterium acnes* | *-* |
| P24 |  | - | - | *Micrococcus luteus* |
| P25 |  | *-* | *Cutibacterium acnes* | - |
| P26 | VD | *Micrococcus luteus,* *Bacteroides sp.,* *Clostridium innocuum,* *Collinsella aerofaciens* | *Bacteroides sp.,* *Clostridium innocuum,* *Collinsella aerofaciens,* *Enterococcus durans,* *Parabacteroides merdae,* *Corynebacterium simulans* | *Bacteroides sp.,* *Clostridium innocuum,* *Collinsella aerofaciens,* *Enterococcus durans,* *Corynebacterium sp.,* CN-staphylococci*,* *Coprobacillus cateniformis* |
| P27 |  | CN-staphylococci*,* *Dermabacter jinjuensis, Moraxella osloensis,* *Corynebacterium tuberculostearicum,* *Cutibacterium avidum,* *Bacteroides diastonis,* *Lactobacillus crispatus* | CN-staphylococci*,* *Dermabacter jinjuensis,* *Corynebacterium sp.,* *Micrococcus luteus,* *Micromonas micros,* *Facklamia hominis,* *Anaerococcus octavius* | *Corynebacterium tuberculostearicum* |
| P28 |  | CN-staphylococci*, Escherichia fergusonii,* *Cutibacterium sp.,* *Corynebacterium tuberculostearicum,* *Clostridium innocuum,* *Streptococcus sp.,* *Prevotella bivia* | CN-staphylococci*,* *Staphylococcus aureus,* *Streptococcus anginosus,* *Micrococcus luteus,* *Bifidobacterium spp* | *Cutibacterium acnes* |
| P29 |  | *Lactobacillus crispatus,* *Staphylococcus haemolyticus,* *Corynebacterium sp.,* *Streptococcus salivarus,* *Collinsella aerofaciens,* *Bacteroides sp.,* *Coprococcus comes* | *Bifidobacterium sp., Odoribacter splanchnicus,* *Collinsella aerofaciens,* *Lactobacillus crispatus,* *Bacteroides faecis* | *Lactobacillus crispatus* |
| P30 |  | *Actinomyces urogenitalis,* *Bacteroides fragilis,* *Tyzerella nexilis,* *Prevotella bivia,* *Fusobacterium nucleatum,* *Desulfovibrio desulfuricans,* *Peptoniphilus sp.,* *Escherichia fergusonii,* *Streptococcus anginosus,* *Fenollaria massiliensis,* n. d. | *Staphylococcus aureus,* *Escherichia fergusonii,* n. d.*,* *Prevotella sp.,* *Clostridium innocuum,* *Streptococcus mitis,* *Peptoniphilus lacrimalis,* *Actinomyces urogenitalis,* *Anaerococcus lactolyticus* | *-* |
| P31 |  | *Lactobacillus gasseri,* *Corynebacterium sp.,* *Gardnerella vaginalis,* n. d. | *Staphylococcus lentus, Corynebacterium sp.* | *Lactobacillus gasseri,* *Gardnerella vaginalis,* n. d. |
| P32 |  | *Streptococcus oralis,* *Peptoniphilus sp., Fenollaria massiliensis,* *Collinsella aerofaciens,* *Bifidobacterium adolescentis,* n. d. | *Streptococcus oralis,* *Parabacteroides merdae,* *Fenollaria massiliensis,* *Cutibacterium acnes,* *Collinsella aerofaciens,* *Levyella massiliensis,* *Corynebacterium sp.,* *Finegoldia magna,* *Varibaculum cambriense* | *-* |
| P33 |  | *Streptococcus oralis,* *Staphylococcus epidermidis,* *Bacteroides vulgatus,* *Metaprevotella massiliensis,* *Prevotella sp.,* *Propionimicrobium lymphophilum,* *Levyella massiliensis,* *Peptoniphilus grossesensis,* *Porphyromonas assacharolytica,* *Schaalia turicensis,* n. d. | *Staphylococcus epidermidis, Corynebacterium amycolatum,* *Actinomyces neuii,* *Bifidobacterium sp.,* *Prevotella sp.,* *Cutibacterium acnes,* *Peptoniphilus sp.,* *Propionimicrobioum lymphophilum,* n. d. | *-* |
| P34 |  | CN-staphylococci*, Escherichia fergusonii, Corynebacterium amycolatum,* *Lactobacillus sp.,* *Anaerococcus obesiensis,* *Peptoniphilus sp.,* *Finegoldia magna,* *Cutibacterium acnes* | *Staphylococcus haemolyticus,* *Lactobacillus crispatus,* *Peptoniphilus lacrimalis,* *Prevotella colorans,* *Kocuria varians* | - |
| Negative controls |  | *Staphylococcus hominis, Kocuria rhizophila, Nocardiopsis synnemataformans, Cutbacterium acnes, Bacillus firmus, Micrococcus spp., Staphylococcus auricularis, Staphylococcus epidermis, Staphylococcus haemolyticus, Moraxella osloensis* | | |
