# Supplementary figures and images for: Evidence for contamination as the origin for bacteria found in human placenta rather than a microbiota
Source: PLoS One. 2020 Aug 10;15(8):e0237232. doi: 10.1371/journal.pone.0237232 (PMC7416914; doi:10.1371/journal.pone.0237232)

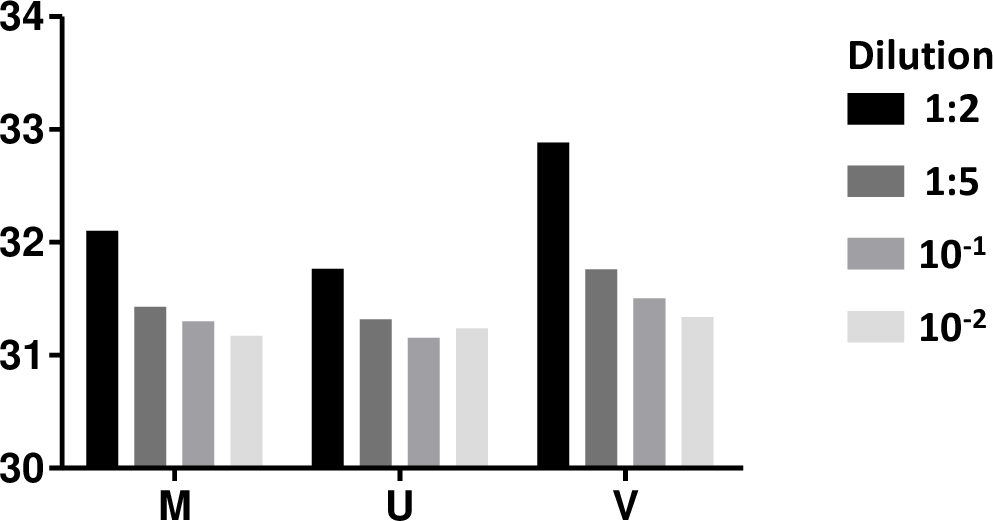

Supplement: S1 Fig — As the dilution factor of extracted DNA increased, the detection of control DNA was earlier indicating presence of inhibitors in extracted DNA from all areas sampled. The 1:5 dilution seemed to eliminate most of inhibitors since it did not drastically vary with higher dilution factors. M: fetal membranes; U: umbilical cord; V: chorionic villi. (TIF) [file pone.0237232.s002.tif]

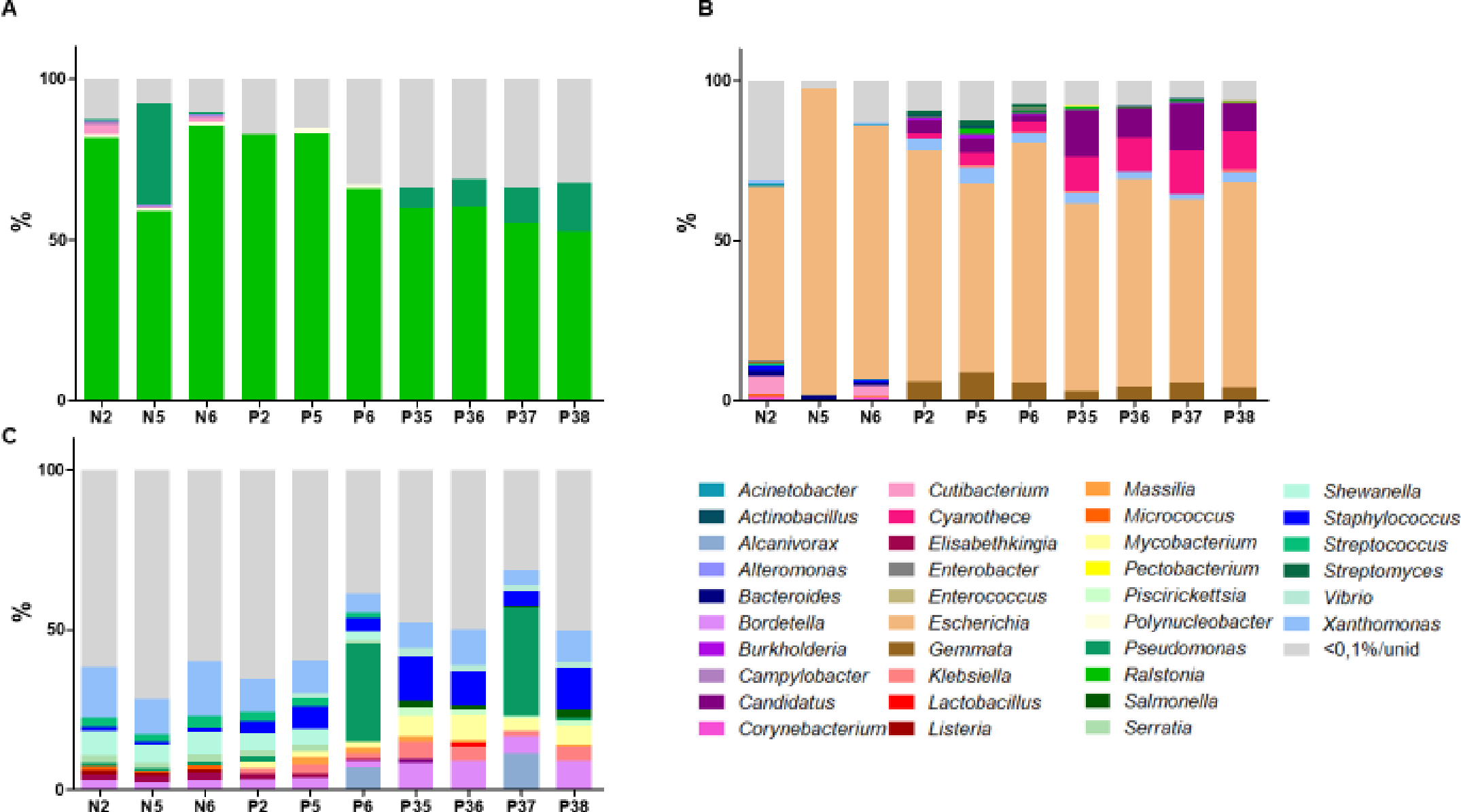

Supplement: S2 Fig — N: negative control; P: placental sample. (TIF) [file pone.0237232.s003.tif]
